# Supplementary figures and images for: Impact of high platelet turnover on the platelet transcriptome: Results from platelet RNA-sequencing in patients with sepsis
Source: PLoS One. 2022 Jan 27;17(1):e0260222. doi: 10.1371/journal.pone.0260222 (PMC8794123; doi:10.1371/journal.pone.0260222)

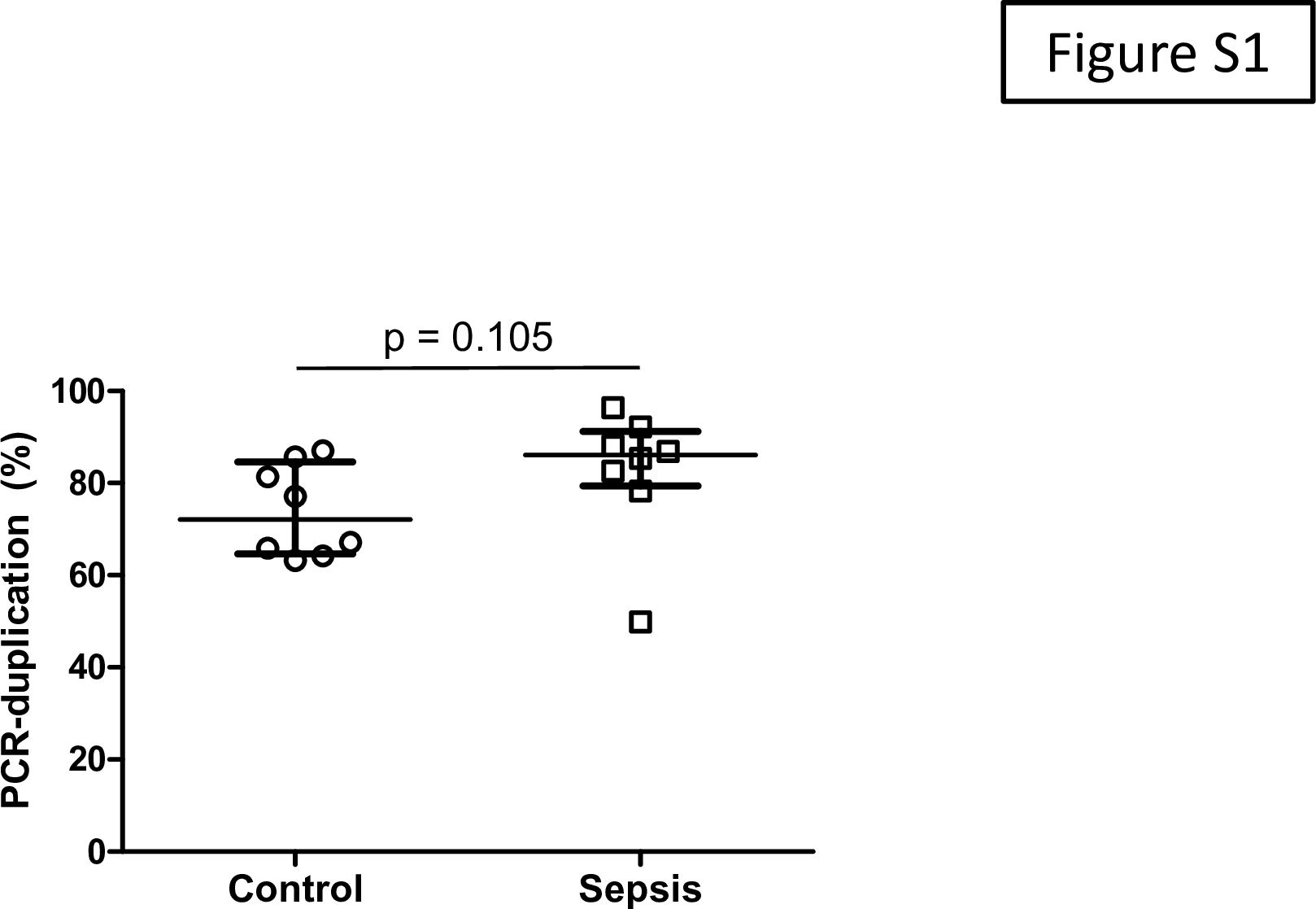

Supplement: S1 Fig — PCR duplication as identified and removed by Je-MarkDuplicates in individual samples. P value from Mann-Whitney U test. (TIF) [file pone.0260222.s001.tif]

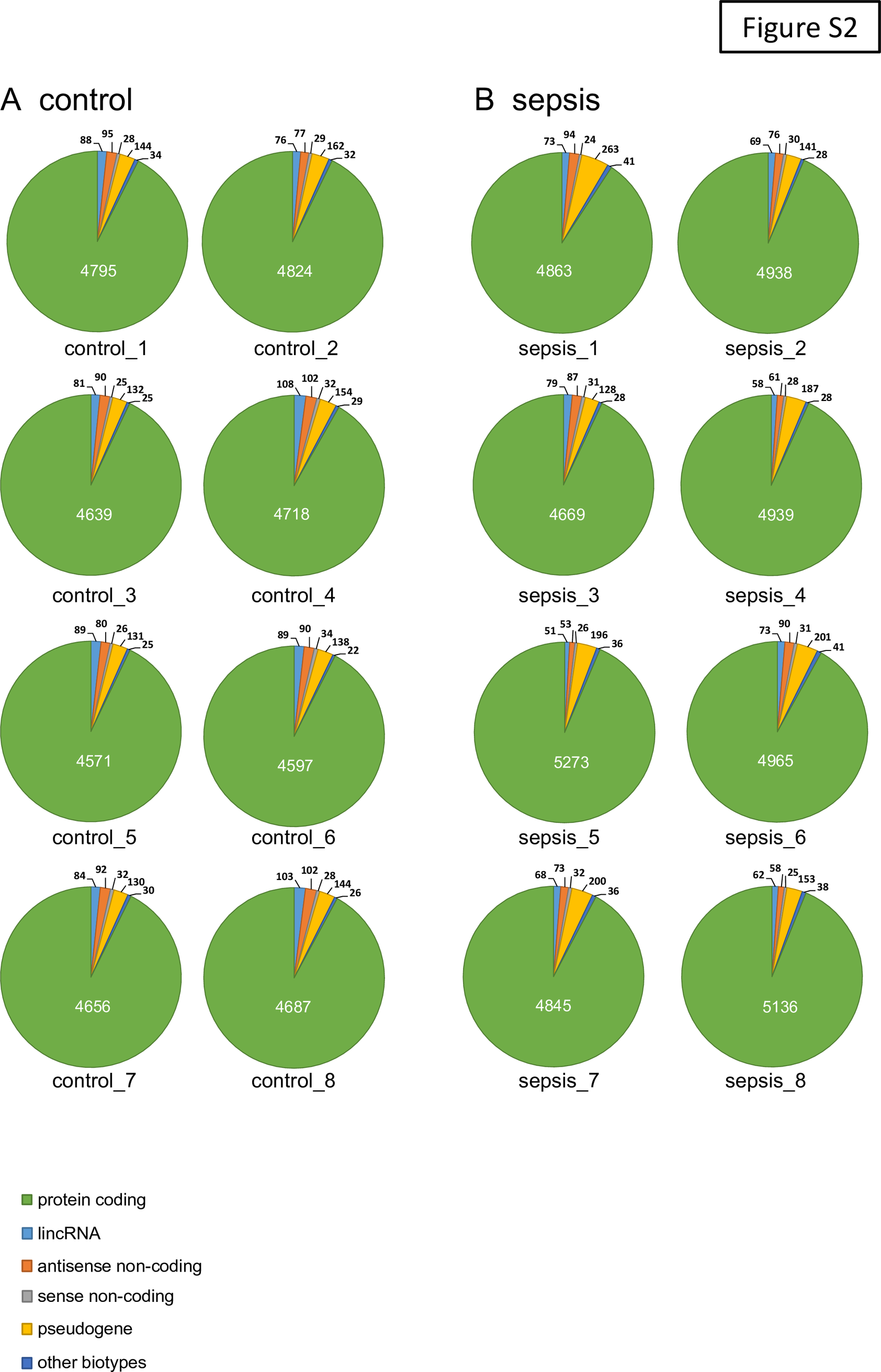

Supplement: S2 Fig — For each individual patient, the number of transcripts with a normalized count of ≥ 10 is displayed according to their gene biotype. (TIF) [file pone.0260222.s002.tif]

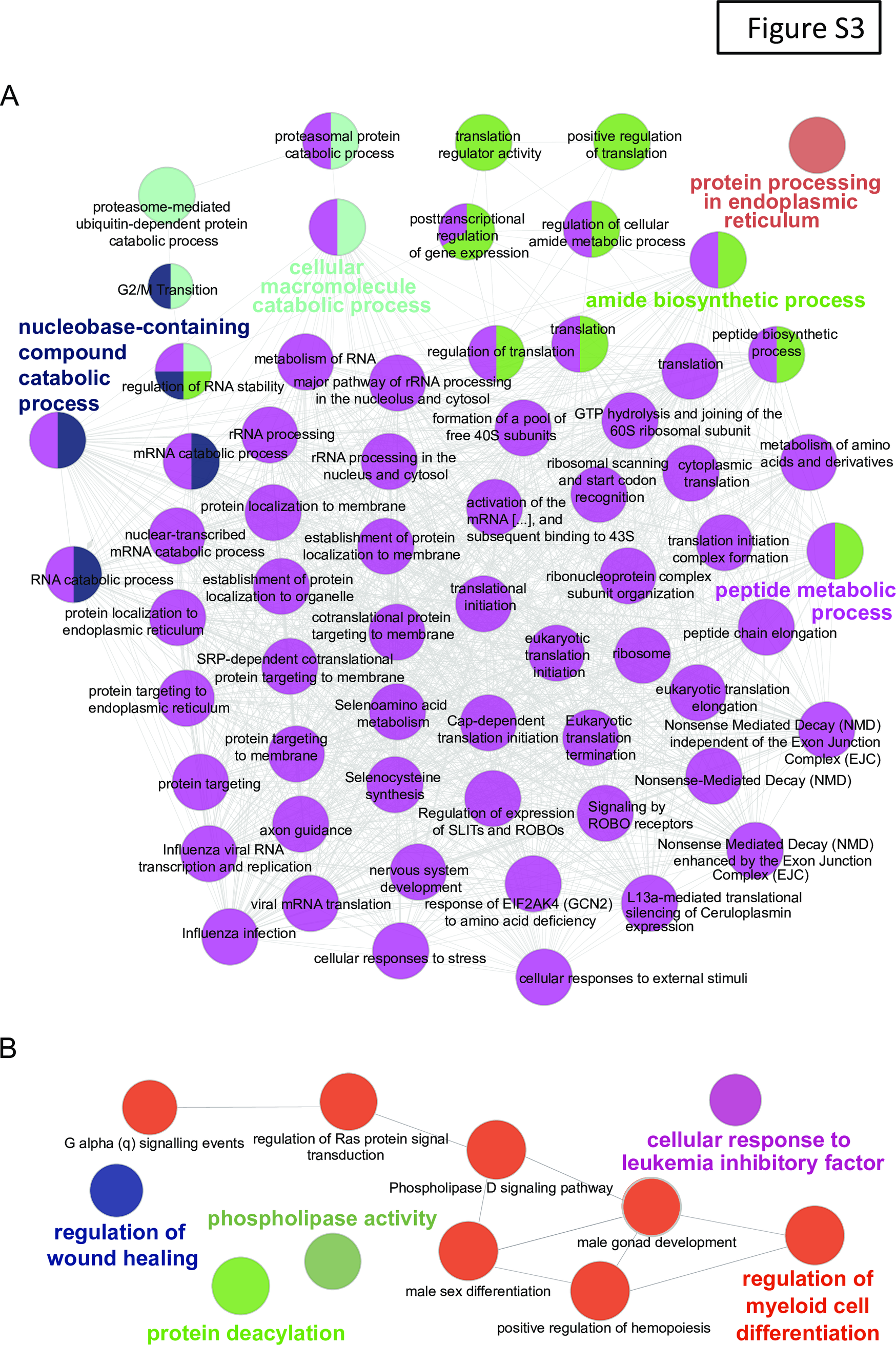

Supplement: S3 Fig — Functional annotation of differentially expressed genes is analysed by ClueGO, a Cytoscape plugin, for upregulated genes (A) and downregulated genes (B) with similar parameters using Gene Ontology (GO), Reactome and KEGG terms. Terms with highest significance within one network are spelled in the respective colour. (TIF) [file pone.0260222.s003.tif]
