# Supplementary material for: Impact of high platelet turnover on the platelet transcriptome: Results from platelet RNA-sequencing in patients with sepsis
Source: PLoS One. 2022 Jan 27;17(1):e0260222. doi: 10.1371/journal.pone.0260222 (PMC8794123; doi:10.1371/journal.pone.0260222)
Supplement: Attachment — Submitted filename: Point to point response to reviewers comments.docx [file pone.0260222.s006.docx]

We wish to thank the reviewer 1 for her/his detailed comments and suggestions that helped to tremendously improve the manuscript.

Nührenberg et al. compared the transcriptome profile of 8 patients with sepsis and 8 control patients with stable CAD, in order to evaluate the specific profile of immature platelets, abundant in patients with sepsis. The study is well conducted, and the manuscript well written.

However, here are a few minor comments that should be addressed in order to improve the manuscript:

1. Add DEG to the abbreviations and meaning in main text.

We noticed that the abbreviation 'DEG' was only used once. Thus, we chose not to use this abbreviation and rephrased the text of the methods section accordingly:

*Methods section, paragraph ' RNA-sequencing data analysis' was rephrased as followed:*
Genes ~~DEGs~~ were considered ~~significant~~ differentially expressed if p-adj (~~adjusted~~ p value corrected by Benjamini-Hochberg procedure) was < 0.05 from DESeq2 and log2 fold change was > ± 1.5.

2. Typo at: 'Gene Set Enrichment Enalysis'

We thank the reviewer to have noted the typo and corrected the error.

*In the methods section, the following phrase was changed:*
For evaluation of activating and inhibiting pathways in platelets [21], Gene Set Enrichment ~~E~~Analysis (GSEA) [22] was performed.

3. Regarding the distinct profiles of Sepsis_2 / 3 and Control_2, is there anything particular
about these participants that could explain the peculiar transcriptome profiles (e.g.
medication)?

We reviewed all available characteristics including medication of samples sepsis_2/3 and control_2 with regard to possible specific characteristics that might explain their distinct gene expression profiles not clustering together with their respective cohort samples. However, we did not detect any characteristics that were in congruent contrast to the remaining samples. Hence, we need to conclude that there is considerable heterogeneity in clinical samples that cannot be explained by the available characteristics.

*In the discussion section, the following phrase was added in the 'Limitations' paragraph:*
Still, as it is not unusual with clinical samples as compared to samples from standardised experimental conditions, we observed considerable heterogeneity between the samples that could not be explained by available patient characteristics.

4. Please add information on the sex of participants.

We apologize for not having provided this information in Table 1. All patients were male; we added this information in Table 1 and in the results section.

*In the Results section, the following phrase was added in the 'Baseline and laboratory characteristics' paragraph:*
Considering demographic characteristics, septic (n=8) and control patients (n=8) were all male and were well matched (Table 1).

|  | **Entire cohort  (n = 16)** | **Control (n = 8)** | **Sepsis (n = 8)** | **p value (sepsis vs. control)** |
| --- | --- | --- | --- | --- |
| **Clinical parameters** |  |  |  |  |
| Age (years) | 75 [69 - 78] | 76 [72 - 80] | 72 [62 - 77] | 0.127 |
| Female sex | 0 (0%) | 0 (0%) | 0 (0%) | 1 |
| Body mass index (kg/m²) | 29 [27 - 34] | 29 [28-34] | 28 [25 - 32] | 0.328 |

5. Was there any adjustment (age, sex, PCs, …) performed for the analyses?

We thank the reviewer for raising this important point. We did not perform adjustment in the analyses for differential transcript expression for several reasons:
i) the cohort was small, ii) variables that were not possibly influenced by sepsis were not significantly different between the two groups, iii) and all variables with significant differences between the two groups could be related to the presence of sepsis, with possible collinearity to sepsis.

*In the Results section, the following phrase was added in the paragraph '* *Differentially expressed genes in platelets from patients with sepsis':*
No adjustment for clinical characteristics with significant differences was performed since all these variables are directly affected by sepsis.

6. Please add sample clustering to Fig 5B

As indicated by the sample caption in Figure 5B, no sample clustering was performed in the heatmap. We decided to omit sample clustering since the heatmap represents only transcripts differentially expressed between sepsis and control samples according to Deseq2 analysis. Despite this preselection, sample clustering would result in clustering of sample 'control_2' with the sepsis samples. This underlines that there is considerable heterogeneity in clinical samples. We added this fact in the manuscript text and highlighted in the figure legend that only transcript clustering was performed.

*In the Results section, the following phrase was added in the paragraph '* *Analysis of prespecified gene sets of platelet activation signaling:*
If sample clustering were performed, sample Control_2 would cluster with the sepsis samples, despite the restriction to differentially expressed genes (data not shown).

*The legend of Figure 5B was modified as followed:*
(B) Differential gene expression illustrated by a heatmap with transcript clustering.

7. Consider adding the normalized abundances as a supplementary table

As proposed by the reviewer, we provide a table with the normalised counts from Deseq2 as a supplementary table S2.

8. Consider adding DEG results as a supplementary table (we can’t see individual genes on the heatmap)

In supplementary table S2, we also provide a sheet with DEG results displaying the 524 transcripts identified as upregulated in sepsis and the 118 transcripts identified as downregulated in sepsis.

9. The p-value adjustment procedure is not defined (FDR? Bonferroni?)

We apologize for not having stated the p-value adjustment procedure. The default option of Deseq2 algorithm was used, which is a Benjamini-Hochberg procedure (FDR). We added this information to the methods section.

*Methods section, paragraph ' RNA-sequencing data analysis' was rephrased as followed:*
Genes ~~DEGs~~ were considered ~~significant~~ differentially expressed if p-adj (~~adjusted~~ p value corrected by Benjamini-Hochberg procedure) was < 0.05 from DESeq2 and log2 fold change was > ± 1.5.

10. The alignment of Fig 3 C/D and Fig 5 C/D is a bit confusing. Consider aligning the 0 value of red/blue tracks. Also it’s not clear why abundances can be negative, I’m not sure the authors specified the normalization/scaling procedure. If not, please add it.

We agree with the reviewer that Figures with traces from the integrated genome viewer to illustrate genomic transcript expression may be confusing. In Figure 3C/D, both groups were aligned to the 0 value. The negative value as it is visualized in the integrated genome viewer to indicate a reverse genomic orientation of the respective gene, was changed to positive values and the genomic orientations of the genes were added. For space reasons, the respective opposite strands are not shown. In Figure 5 C/D, forward and reverse strands are shown for each gene to improve clarity. Transcripts were normalized by Fragments Per Kilobase of transcript, per Million mapped reads (FPKM).

*In the* *Methods section, paragraph ' RNA-sequencing data analysis' the following phrase was added:*
Transcripts visualised in IGV were normalised by Fragments Per Kilobase of transcript, per Million mapped reads (FPKM).

*Figure 3 C/D was changed as followed:*

*Figure 5 C/D was changed as followed:*

11. Please add a more complete description to the GSEA figures (Fig 7 and 8). Readers won’t understand the output of GSEA if you don’t describe it. And please define what is the “core enrichment”.

As suggested, we added a more complete description to the GSEA figure legends. Also, 'core enrichment' was explained in the manuscript text and in the figure legends.

*In the Results section, the following phrases were added in the paragraph 'Differentially expressed genes in platelets from patients with sepsis':*
The normalised enrichment score (NES) represents the primary metric since it allows comparison between gene sets by taking the gene set size and correlations between the expression data and the gene sets into account [24].

Core enrichment comprises the leading edge subset of genes that add most to the enrichment score, being ranked at the top of the gene set before a positive peak enrichment score [24].

*In the Figure legends section, the following phrase was added in the legends of figures 7 and 8:*
The normalised enrichment score (NES) represents the primary metric since it allows comparison between gene sets by taking the gene set size and correlations between the expression data and the gene sets into account [24].

*In the Figure legends section, the following phrase was added in the legend of figure 7:*
Core enrichment comprises the leading edge subset of genes that add most to the enrichment score, being ranked at the top of the gene set before a positive peak enrichment score [24].

12. Consider adding a brief statement with quality metric results to demonstrate the benefits of UMI to remove PCR amplification biases.

We thank the reviewer for pointing to this important methodological aspect of our study. We have now added respective phrases in the results section describing the results and benefits of removing PCR duplicates and prepared a figure that may be added as a supplemental figure.

*Supplemental Figure S1 was added:*

*The following phrase was added to the results section, paragraph 'Global transcript distribution and purity of ribosome-depleted platelet RNA-sequencing':*
Duplicate removal by Je [14] identified a large proportion of PCR duplicates among mapped reads - ranging from 49.9% to 96.4% (supplemental Figure S1).

*The following phrase was added to the results section, paragraph 'Differentially expressed genes in platelets from patients with sepsis':*
If a similar analysis with no PCR duplicate removal was performed, only 68 transcripts would have been considered as upregulated and 55 transcripts as downregulated (data not shown).

*The following Figure caption and legend was added to the Figure Legends section:*
**Figure S1: Percentage of PCR duplication in individual samples**

PCR duplication as identified and removed by Je-MarkDuplicates in individual samples. P value from Mann-Whitney U test.

We are grateful for the constructive comments of reviewer 2 that helped to strengthen the manuscript.

The manuscript investigates by RNA sequencing in patient platelets during sepsis and compares them to control patients with stable coronary disease. Very laudable study and interesting data.

1. The immature platelet fraction (IPF) was significantly different and even the ranges are given. Please comment how the really low p-value is possible, may be this is due to the confidence intervalls given (so 2 standard deviations) in the abstract? If so, please mention in the abstract shortly (e.g. +/- 2s)

We apologize for possible ambiguity regarding the confidence intervals in the abstract that may have made it difficult to comprehend the low p value for the comparison of IPF values between the two groups. We specified also in the abstract that continuous variables are reported as median with interquartile range.

*Abstract, paragraph ‘results’ was changed to read:*

IPF (median [interquartile range]) was significantly elevated in sepsis patients (6.4 [5.3 – 8.7] % vs. 3.6 [2.6 – 4.6] %, p=0.005).

2. Data availability: the EMBL nucleotide archive number is given, however, please comment briefly, in which state the archive is (do we have the tag counts for each mRNA available for the user or do we have just the raw data etc.). To be useful for the future reader, it should be made sure that the mapped reads are available to the reader, too.

As recommended by ENA, we uploaded all files in BAM format. Additionally, to ensure reproducibility with regard to the PTESfinder analysis, we also uploaded fastq files. We added this specification to the methods section. Since no specific reviewer access is possible with sequencing experiments in ENA due to cross-referencing with SRA, we made the data public to allow access for the reviewer (https://www.ebi.ac.uk/ena/browser/view/ PRJEB45262).

*Methods section, paragraph 'Data availability' was changed to read:*
The RNA-seq data presented in this manuscript have been deposited under the accession number PRJEB45262 in the European Nucleotide Archive as mapped BAM and raw FASTQ files (ENA, <https://www.ebi.ac.uk/ena/browser/view/PRJEB45262>).

3. Results: In Figure 4 (PCA) we see in the left panels that the sample Sepsis2 clusters with the controls. Please explain how this is possible. Maybe a bad sample. Would the removal of this sample "sepsis2" from the analysis increase the separation between both groups? If so, please correct your analysis accordingly.

We understand that it seems surprising how a sepsis sample may cluster with the control samples. To exclude the possibility of a ‘bad sample’, RNA amount and libraries were controlled after RNA extraction respectively library preparation. No abnormalities were seen in this regard. Also, as suggested by reviewer 1, we reviewed all available characteristics including medication of samples sepsis_2/3 and control_2 with regard to possible specific characteristics. However, we did not detect any characteristics that were in congruent contrast to the remaining samples. Hence, we need to conclude that there is considerable heterogeneity in clinical samples that cannot be explained by the available characteristics.

*In the discussion section, the following phrase was added in the 'Limitations' paragraph:*
Still, as it is not unusual with clinical samples as compared to samples from standardised experimental conditions, we observed considerable heterogeneity between the samples that could not be explained by available patient characteristics.

4. Discussion: I agree to the limitations and that nevertheless hypotheses can be generated. However, then please clearly phrase your 3-4 main hypotheses your data generated just before this section "limitations".

As proposed by the reviewer and in order to avoid redundancy, we restructured the discussion section and listed our main hypotheses before the limitations section.

*In the discussion section, the following phrases were moved before the 'Limitations' paragraph:* Overall, the present data suggest that levels of reticulated platelets may become elevated due to various reasons - such as increased consumption or reactive thrombopoiesis - but their transcriptomic alterations will rather depend on the underlying disease condition. Further studies examining sorted reticulated platelets or platelet ribosome profiling in disease conditions different from profound, systemic inflammation may shed further light on disease-specific translation in platelets.

*In the discussion section, the following phrases were added before the 'Limitations' paragraph:*

In the long term, selective inhibition of platelet-specific translation could represent an attractive therapeutic target, provided that such translation has relevant disease-modifying impact.

5. Figure legend: the comparisons done should be made more clear in the legend.

We apologize if there is ambiguity to the comparisons done. As suggested, we outlined the comparisons performed in the Figure legends.

*In the Figure legends section, the paragraph relating to Figure 1 was changed as followed:*

Comparison between control patients (transparent bars) and patients with sepsis (filled bars) in A-F. Platelet […]

*In the Figure legends section, the paragraph relating to Figure 2 was changed as followed:*

Comparison between control patients (transparent bars) and patients with sepsis (filled bars) in A and B. PTES transcripts or putative circular RNA transcripts were identified and normalised for each patient to (A) the overall number of mapped reads or (B) to the sum of canonical reads as identified by PTESfinder. Filling colour of data points indicates individual samples within the respective groups, boxes with inner line display interquartile range with median, whiskers minimum and maximum of values. P values from Mann-Whitney U test. (C) Volcano plot of differential expression showing absence significant differences between control patients and patients with sepsis. Note the different y-axis as compared to Figure 5. […]

6. Fig. 3 legend ... all samples (give short description of the sample, I think this is sepsis and coronar disease patients and platelets taken from both groups)

As proposed by the reviewer, we rephrased the figure legend to improve clarity.

*In the Figure legends section, the paragraph relating to Figure 3 was changed as followed:*

Illustration of global transcript expression in platelets and purity of platelet RNA-Seq separately for each group. A and B show c~~C~~onsistently expressed transcripts, defined as ~~expression present in all samples and~~ a median normalised count of ≥ 10 and expression across all 8 samples of each cohort, in control patients (A) and patients with sepsis (B). Genome Browser views of Protein Tyrosine Phosphatase Receptor Type C [PTPRC] gene / CD45 gene (C) and Purinergic Receptor P2RY12 [P2RY12] (D) expression. Individual samples from patients with sepsis are shown in the top part (indicated by left-sided red bar), individual samples from control patients are shown in the lower part (indicated by left-sided blue bar).

7. Fig. 6-8 legend: again make really clear what is compared against what, for instance in the figure title

As suggested, we added more detailed descriptions to the figure titles of Figures 6 - 8.

*In the Figure legends section, the figure title relating to Figure 6 was changed as followed:*
Figure 6: Functional annotation of genes differentially expressed ~~genes~~in platelets from septic patients versus platelets from control patients

*In the Figure legends section, the figure title relating to Figure 7 was changed as followed:*
Figure 7: Gene Set Enrichment Analysis with predefined gene sets related to platelet activation identifies two gene sets enriched in the platelet transcriptome from patients with sepsis compared to control patients.

*In the Figure legends section, the figure title relating to Figure 8 was changed as followed:*
Figure 8: ~~Comparison of g~~Gene expression in platelets from patients with sepsis and control patients is compared with platelet transcriptome changes in other patients with sepsis, patients with COVID-19 and reticulated platelets by Gene Set Enrichment Analysis

We are thankful to reviewer 3 for his valuable comments that helped to profoundly improve the manuscript.

1. This paper looks at 8 patients with sepsis and 8 without. One assumes that the exclusion criteria for the sepsis group was also true in the control group as these are stable patients - but it would be good to explicitly state this as it reduces the possibility of differences between groups.

We apologize for the ambiguity concerning the exclusion criteria in the control group. As suggested, we rephrased the respective sentences in the methods section.

*In the Methods section, the paragraph 'Subjects, inclusion and exclusion criteria, blood sampling' was changed as followed:*
Inclusion criteria for sepsis were a CRP-level > 5 mg/dl in combination with a clinically manifest infection, a procalcitonin level > 0.05 ng/ml, or positive blood cultures. ~~Exclusion criteria in sepsis were patients receiving platelet or clotting factor transfusions, patients with known platelet disorders or with heparin induced thrombocytopenia, patients undergoing acute or chronic haemodialysis or peritoneal dialysis, and patients with an advanced malignancy.~~

Control patients all had stable coronary artery disease and presented similar biometrical and demographic characteristics as septic subjects, though lacking acute or chronic signs of infection as well as signs of acute illness. Exclusion criteria in all patients were patients receiving platelet or clotting factor transfusions, patients with known platelet disorders or with heparin induced thrombocytopenia, patients undergoing acute or chronic haemodialysis or peritoneal dialysis, and patients with a ~~Exclusion criteria were presence of~~ malignancy or a~~of~~ haematological disorder.

2. I am entirely unclear of the rationale for 8 vs 8 here - please justify this in terms of whether there was a primary hypothesis, or whether the data are purely exploratory. Is there sufficient power here to detect appropriate differences?

We thank the reviewer for raising this important point. While our study was primarily exploratory, our primary hypothesis was to detect differential platelet gene expression by RNA-Seq between patients with sepsis and control patients.

According to RNASeqPower - a Bioconductor /R package for sample size calculation in RNASeq studies (10.18129/B9.bioc.RNASeqPower) - with 8 patients, an α of 0.05, effect sizes we desire to detect of > 2.83 (log2 fold change > 1.5), and coefficient of variation of 0.6, we expect to achieve a power of 0.9.

Also, it has nicely been laid out that DESeq2, the tool we used for assessment of differential gene expression, is adequate to use in this number of samples (PMID 27022035).

We therefore suggest that, while our study was primarily exploratory, sufficient power was given to detect meaningful differences and added these reflections to the methods section.

*In the Methods section, the following phrases were added to the paragraph 'RNA-sequencing data analysis':*
According to RNASeqPower - a Bioconductor /R package for sample size calculation in RNASeq studies [17] - with 8 patients, an α of 0.05, effect sizes we desire to detect of > 2.83 (log2 fold change > 1.5), and coefficient of variation of 0.6, we expect to achieve a power of 0.9. The use of DESeq2, the tool used for assessment of differential gene expression, was considered adequate in this number of samples [18].

*In the Limitations section, the following phrase was added:*

Also, according to sample size estimation, the current number of samples may detect transcriptomic changes to the described extent (log2 fold change of >1.5) at a power of 0.9.

3. Why are the p-values for LV function and peripheral arterial disease missing - surely they are exactly 1.

We apologize for not having provided the exact p-value for impaired left ventricular function and the presence of peripheral artery disease. Indeed, they are both 1.

*Table 1, was changed as followed:*

|  | **Entire cohort  (n = 16)** | **Control (n = 8)** | **Sepsis (n = 8)** | **p value (sepsis vs. control)** |
| --- | --- | --- | --- | --- |
| **Clinical parameters** |  |  |  |  |
| … |  |  |  |  |
| Reduced LV function | 8 (50 %) | 4 (50 %) | 4 (50%) | 1 |
| Peripheral artery disease | 4 (25%) | 2 (25%) | 2 (25%) | 1 |

4. Figures 1 and 2 show box and whisker plots and significance marked with star notation. Please give exact p-values, and annotate the boxes and whiskers with medians and ranges for each group. It is not correct to simply mark the significant results.

As suggested, we now added exact p-values in Figures 1 and 2. Regarding the boxes and whiskers, median with interquartile range is represented by the box and inner line, while the range of values is indicated by the whiskers. We added the respective description to the legend of Figure 2 where it was missing.

*In the Figure legends section, the paragraph relating to Figure 2 was changed as followed:*
Comparison between control patients (transparent bars) and patients with sepsis (filled bars) in A and B. PTES transcripts or putative circular RNA transcripts were identified and normalised for each patient to (A) the overall number of mapped reads or (B) to the sum of canonical reads as identified by PTESfinder. Filling colour of data points indicates individual samples within the respective groups, boxes with inner line display interquartile range with median, whiskers minimum and maximum of values. P values from Mann-Whitney U test.

*Figure 1 was changed to illustrate exact p-values for all comparisons as followed:*

*Figure 2 A and B was changed to illustrate exact p-values for all comparisons as followed:*

5. Figures 2C and D clearly show the results of more than one value per patient - these data are therefore likely to be clustered. How is this clustering taken into account, and why if Mann-Whitney U tests are used elsewhere is not Spearman's rho used in the correlation?

We apologize for not having adequately described the data shown in Figures 2 C and D. Indeed, the data points shown are not values from single patients but mean normalized mRNAs and PTES counts for 1404 expressed PTES transcripts. To this end, we rephrased the results section and the legend of Figure 2 C and D. Also, we know use Spearman’s *ρ* in the correlation as suggested.

*In the Results section, the last phrase of the paragraph relating to PTES transcripts was changed as followed:*

Further evaluation did not show a differential regulation of PTES transcripts in platelets from septic patients (Figure 2C) but a low, yet highly significant correlation ~~of~~ between mean PTES and respective linear transcript~~s~~ expression (Figure 2D).

*In the Figure legends section, the phrasing relating to Figure 2 C and D was changed as followed:*

(C) Volcano plot of adjusted p values from differential expression analysis by edgeR versus log2 fold changes showing absence of significant differences between control patients and patients with sepsis. Note the different y-axis as compared to Figure 5. (D) ~~Pearson~~ Spearman correlation between mean mRNA expression and mean PTES expression levels. Note the off-set x-axis, limiting PTES counts to values > 8. Lower values were filtered out for low expression in differential expression analysis.

6. Again in Figure 3A, B to what extent are the features seen here equally distributed among patients?

In Figure 3 A and B, we intended to demonstrate the overall distribution of consistently expressed transcripts, separately for each group. This way, a large difference in the distribution of expressed transcripts would be visible. To illustrate the point that is raised by the reviewer, i.e. how much variation is present between the individual patients, we analysed the distribution of transcripts with a normalised count of ≥ 10 for each patient separately. If the reviewer agrees, we would add these results to the manuscript and as a supplemental figure.

*In the Results section, the paragraph relating to global transcript distribution was changed as followed:*

In both groups, most expressed transcripts were protein-coding genes and only minor changes occurred in the number of consistently expressed genes, defined as expression present in all samples and a median normalised count of ≥ 10 (Figure 3A, 3B). Also, patterns of transcript distribution were largely similar across all samples when a normalised count ≥ 10 was used as cut-off for transcript expression (Figure S2).

*The following Figure caption and legend was added to the Figure Legends section:*
**Figure S2: Number of expressed transcripts per individual patient**

For each individual patient, the number of transcripts with a normalized count of ≥ 10 is displayed according to their gene biotype.

*Supplemental Figure S2 was added:*

7. To make it clear that the data is anonymised, it might be better not to use the name of the first author in the legends to figure 8.

As suggested, we omitted the names of the first authors in Figure 8.

*Figure 8 was changed as followed:*

8. Please explore the question of whether non-significant results mean evidence of absence of difference or merely absence of evidence. It is not clear here the extent to which dichotomisation of p-values identifies the important aspects here and how the issue of multiple testing is allowed for.

We agree with the reviewer’s general statement that it is important to reflect on these aspects. Hence, we discussed in the limitations section that our study should be seen as hypothesis-generating. Also, since we cannot disprove that reticulated platelets may have influenced platelet-specific gene expression in our study, we mentioned this aspect and suggested ribosomal foot printing in different disease conditions to shed further light on translation in reticulated platelets. Concerning the issue of multiple testing, the method used for detection of differential gene expression – Deseq2 – provides adjusted p values by the Benjamini-Hochberg procedure to allow for multiple testing in RNASeq experiments (see reviewer 1, point #9).

*Methods section, paragraph ' RNA-sequencing data analysis' was rephrased as followed:*
Genes ~~DEGs~~ were considered ~~significant~~ differentially expressed if p-adj (~~adjusted~~ p value corrected by Benjamini-Hochberg procedure) was < 0.05 from DESeq2 and log2 fold change was > ± 1.5.
